# *Escherichia coli* Bloodstream Infections in Patients at a University Hospital: Virulence Factors and Clinical Characteristics

**DOI:** 10.3389/fcimb.2019.00191

**Published:** 2019-06-06

**Authors:** Ana Paula Daga, Vanessa Lumi Koga, João Gabriel Material Soncini, Caroline Martins de Matos, Marcia Regina Eches Perugini, Marsileni Pelisson, Renata Katsuko T. Kobayashi, Eliana Carolina Vespero

**Affiliations:** ^1^Laboratory of Clinical Microbiology, Department of Pathology, Clinical and Toxicological Analysis, Health Sciences Center, State University of Londrina, Londrina, Brazil; ^2^Laboratory of Microbiology, Department of Microbiology, State University of Londrina, Londrina, Brazil

**Keywords:** ExPEC, bloodstream infection, phylogenetic group, virulence factors, ESBL, CTX- M-15

## Abstract

Extraintestinal pathogenic *Escherichia coli* (ExPEC) isolates are responsible for many bloodstream infections. The aim of this study was to characterize *E. coli* isolated from the bloodstreams of patients (*n* = 48) at the University Hospital in Brazil. Epidemiological data were obtained through the analysis of medical records and laboratory tests. By PCR analysis, we investigated the presence of virulence factors (VFs), pathogenicity islands (PAIs), extended-spectrum β-lactamase (ESBL), phylogenetic classifications (A, B1, B2, C, D, E, and F) and molecular genotype by enterobacterial repetitive intergenic consensus-polymerase chain reaction (ERIC-PCR). The mortality analysis showed that 33.3% of the deaths were associated with bacteraemia due to *E. coli* infections; in addition, an age between 60 and 75 years (*p* < 0.001; OR = 6.3[2.1–18.9]) and bacteraemia with an abdominal origin (*p* = 0.02; OR = 5[1.2–20.5]) were risk factors for the severity of the infection. Additionally, the presence of the *afa* gene was associated with mortality due to *E. coli* bacteraemia (*p* = 0.027; OR = 11.4[1.5–85.7]). Immunosuppression (27.1%), intestinal diseases (25.0%) and diabetes (18.8%), were prevalent among patients, and most of the bacteraemia cases were secondary to urinary tract infections (50.0%). The serum resistance gene *tra*T was present in 77.1% of isolates, group capsular 2 (*kpsMT* II) was present in 45.8% and the K5 capsule was present in 20.8% of isolates. The isolates also showed a high prevalence for the siderophore yersiniabactina (*fyu*A) (70.8%) and PAI IV_536_ (77.1%). Phylogenetic analysis showed that group B2 (45.8%) was the most prevalent, and was the phylogroup that had a higher prevalence of VFs and PAIs. However, in this study, a considerable number of isolated bacteria were classified as group B1 (18.8%) and as group E (14.6%). Eight (16.7%) isolates were resistant to third and fourth generation cephalosporin and group CTX-M-1 (CTX-M-15) was the most prevalent ESBL type. The molecular genotyping showed two clonal lineages and several isolates that were not related to each other. This study provides additional information on the epidemiological and molecular characteristics of *E. coli* bloodstream infections in Brazil.

## Introduction

*Escherichia coli* is the gram-negative organism most frequently isolated in adult patients with bacteraemia (Mora-Rillo et al., 2015) and in severe cases it may lead to death (Owrangi et al., 2018). The rates of bacteraemia have increased steadily in recent years (De Kraker et al., 2013; Miajlovic et al., 2016). In general, *E. coli* are a part of the normal commensal gut microbiota of healthy human populations (Köhler and Dobrindt, 2011; Micenková et al., 2017). However, some strains can cause intestinal or extraintestinal infections due to specific virulence factors (VFs) (Burdet et al., 2014; Usein et al., 2016).

Isolates that are capable of gaining access to and surviving in the bloodstream are known as extraintestinal pathogenic *E. coli* (ExPEC) (Russo and Johnson, 2003) and cause a variety of infections, including urinary tract infections (UTI), sepsis, and neonatal meningitis (Ron, 2010; Mora-Rillo et al., 2015). The most common extra-intestinal site colonized by these bacteria is the urinary tract, which in turn, is a common source for bloodstream infections (Micenková et al., 2017).

According to the phylogenetic classification, *E. coli* was divided into 7 groups (A, B1, B2, C, D, E, and F). The pathogenic groups of the ExPEC strains generally belong to the groups B2 and D, and the commensal strains that survive in the intestines, i.e., non-pathogenic strains, are generally included in groups A or B1 (Clermont et al., 2013).

ExPEC strains have several virulence factors (VFs) that may play a role in infection by enabling the bacterial cells to colonize the host and disseminate. VFs are either encoded on the bacterial chromosome, where they are usually located within pathogenicity islands (PAIs), or on plasmids (Dale and Woodford, 2015); these VFs include adhesion molecules, iron acquisition systems, host defense-subverting mechanisms, and toxins. Several VFs have been associated with bloodstream infections (Lefort et al., 2011; Mora-Rillo et al., 2015). However, predictions of the initial severity and outcome based on bacterial VFs alone are not entirely accurate, and the consideration of host determinants, including underlying diseases, facilitates such predictions (Jauréguy et al., 2007).

When the infection occurs, the host immune system responds to eliminate the infectious agents (Diacovich and Gorvel, 2010). The progression of infection is mainly associated with the bacterial capability to survive such defenses. In addition, bacterial virulence properties influence the severity and extent of the infection (Lefort et al., 2011; Owrangi et al., 2018). The presence of *E. coli* in the bloodstream can result in the induction of a vigorous host inflammatory response that lead to sepsis, which is associated with high morbidity and mortality (Russo and Johnson, 2003; Miajlovic and Smith, 2014).

In addition, the rates of multidrug resistant (MDR) *E. coli* infections are on the rise. In particular, the predominant ExPEC global lineage sequence type (ST) 131 is frequently associated with fluoroquinolone resistance and the production of extended-spectrum β-lactamases (ESBLs) (Shaik et al., 2017), and the treatment of infections caused by ExPEC has become very challenging due to the emergence of resistance to the first-line and the last-resort antibiotics (Usein et al., 2016; Shaik et al., 2017).

In developed countries, *E. coli* is a very important pathogen in infections of the bloodstream. According to Laupland (2013), *E. coli* was ranked first or second in the incidence of bloodstream infections in countries such as Australia, Canada, Denmark, Finland, Iceland, New Zealand, Sweden, and the USA. However, analyzing the frequency of *E. coli* bloodstream infection at the University Hospital in Brazil, showed that in recent years, *E. coli* was responsible for 4.7% of bloodstream infections. Other studies also showed a low prevalence of *E. coli* bloodstream infections in Brazilian hospitals (Marra et al., 2011; Yokota et al., 2016; Taveira et al., 2017). For this reason, there has been little research regarding *E. coli* bloodstream infection in Brazil. However, knowledge of the impact of VFs, antimicrobial resistance, and host determinants on the severity of *E. coli* bloodstream infections is important for the determination of the epidemiological profiles of bloodstream infections; this could provide targets for specific intervention in the future. The aim of this study was to characterize the VFs, antimicrobial sensitivity profile, and epidemiological data of *E. coli* isolates from the blood of patients at the University Hospital of Londrina.

## Materials and Methods

### Bacterial Isolates

This study included 48 *E. coli* samples that were isolated from the bloodstreams of patients admitted to the University Hospital of Londrina from 2015 to 2017. Haemoculture positive samples were detected by BACTEC™ FX (Becton Dickinson, USA), and aetiological agents were previously identified by the VITEK® 2 system (bioMérieux, USA). Following identification, the isolates were kept in Tryptic Soy Broth with 15% glycerol (−20°C).

### Collected Data

Demographic data were collected from all patients, including age, gender, and comorbidities of medical records provided for consultation by the Medical Archive and Statistics Service (SAME) of the University Hospital of Londrina. The study was approved by the Ethics and Research Committee of the State University of Londrina CAAE 43013315.8.0000.5231.

### Antimicrobial Susceptibility

For the identification of the isolates, the VITEK® 2 GN ID card and the VITEK® 2 AST 239 card were used for antibiogram analyses, which was complemented with the diffusion disk method. All cards are from bioMérieux. The interpretation was performed according to the CLSI 2017 (Clinical and Laboratory Standards Institute) criteria.

### β-Lactamase Characterization

The detection of the *bla*_CTX−M−1_, *bla*_CTX−M−2_, *bla*_CTX−M−8_, *bla*_CTX−M−9_, *bla*_CTX−M−25_ genes was performed by multiplex PCR, as described by Woodford et al. (2006) and *bla*_CTX−M−15_ according to Leflon-Guibout et al. (2004). The PCR reaction was enhanced using the TopTaq® Master Mix Kit (QIAGEN). PCR for the screening of the *bla*KPC gene was performed according to Bradford et al. (2004) (Supplementary Material).

### Phylogenetic Classification

The *E. coli* isolates were classified into 7 phylogenetic groups (A, B1, B2, C, D, E, and F), based on the presence of the genes *chu*A, *yja*A, *arp*A, and *trp*A, and a DNA fragment (TSPE4.C2), which were detected by the PCR method (Clermont et al., 2013) (Supplementary Material).

### Detection of Virulence Factor Genes

The genotypic identification of the main virulence factors in ExPEC was performed using the PCR method. The genes selected are the most frequent in ExPEC and included haemolysins (*hly*A and *hly*F), cytotoxic necrotizing factors (*cnf* 1 and *cnf* 2), colicin V (*cva*C), aerobactin (*iut*A), yersiniabactin (*fyu*A), salmochelin (*iro*N), type 1 fimbrial adhesin (*fim*H), P-fimbriae (*pap*C and *pap*G), S-fimbrial adhesin (*sfa*A and *sfa*S), afimbrial adhesin (*afa*), serum resistance (*iss* and *tra*T), brain microvascular endothelium invasion (*ibe*A), capsules (*kpsMT* K1, *kpsMT* K5, *kpsMT* II, and *kpsMT* III), and an outer membrane protein (*omp*T) (Johnson and Stell, 2000; Koga et al., 2014) (Supplementary Material).

### Detection of Pathogenicity Islands

The presence of sequences associated with seven different PAIs, previously characterized in uropathogenic *E. coli*, was determined (PAI I_536_, II_536_, III_536_, IV_536_, I_CFT073_, II_CFT073_, I_J96_, and II_J96_) (Sabaté et al., 2006; Koga et al., 2014) (Supplementary Material).

### Molecular Genotyping

Enterobacterial repetitive intergenic consensus (ERIC-PCR) was performed as previously described by Versalovic et al. (1991). Analysis of genomic fingerprinting was performed using GelJ v.2.0 software by the Dice similarity method (Heras et al., 2015). Strains were considered genetically related if the similarity index was ≥85%.

### Statistical Analysis

Categorical data are shown frequencies and percentages. The analysis was performed by logistic regression, associated to the selection of stepwise variables, Nagelkerke test to determine the value of Pseudo-R2, and Odds Ratio test associated with the Likelihood test. In addition, the Odds Ratio test associated with the Chi-square test was used. The alpha significance level was 0.05. Data analysis was performed using Statistical Package for Social Sciences (SPSS—IBM Corp., New York, USA), version 20 for Windows.

## Results

### Demographic and Clinical Characteristics

The median age of the 48 patients was 63 years old (range 0–90 years) and 56.2% were male. Twenty-three (47.9%) were aged over 65 years and 4 (8.3%) of patients were aged under 1 year. In this study, the most prevalent comorbidity was systemic arterial hypertension (43.8%), followed by intestinal disease (25.0%), diabetes (18.8%), coronary disease, solid tumor, and renal failure at (12.5%) each, hematological malignancy and cirrhosis at 10.4% each, immunologic disease and viral hepatitis at 8.3% each, and HIV at 6.3% (Table 1).

**Table 1:** Characteristics of patients with *Escherichia coli* bacteremia.

Characteristics	(*n* = 48)
Age (years), median (range)	63 (0–90)
Male gender, n (%)	27 (56.2)
Comorbidities, *n* (%)	
Systemic arterial hypertension	21 (43.8)
Intestinal disease	12 (25.0)
Diabetes	9 (18.8)
Coronary disease	6 (12.5)
Solid tumor	6 (12.5)
Renal failure	6 (12.5)
Hematological malignancy	5 (10.4)
Cirrhosis	5 (10.4)
Immunologic disease	4 (8.3)
Viral hepatitis	4 (8.3)
HIV	3 (6.3)
Origin of Bacteremia, *n* (%)	
Urinary	24 (50.0)
Abdominal	6 (12.5)
Pulmonary	4 (8.3)
Early neonatal sepsis	3 (6.3)
Medical procedure	2 (4.2)
Not defined	9 (18.7)
All-cause mortality, n (%)	24 (50.0)
≤ 14 days	15 (62.5)
15–28 days	3 (12.5)
>28 days	6 (25.0)
Mortality associated with *E. coli*	8 (33.3)
≤ 7 days	8 (100.0)

According to the data from the medical records and laboratory data, 24 (50.0%) patients had bacteraemia from a urinary origin, 6 (12.5%) had abdominal sepsis, 4 (8.3%) had pulmonary sepsis, 3 (6,3%) had early-onset neonatal sepsis, 2 (4.2%) had bacteraemia after a medical procedure, one patient had bacteraemia after amputation of the lower limb, another patient had bacteraemia after a prostate biopsy, and 9 (18.7%) cases of bacteraemia did not have the origin defined. Of the 48 patients studied, half (50.0%) died, and 8 (33.3%) of these patient deaths were related to *E. coli* bloodstream infections (Table 1).

In this study, the univariate statistical analysis showed that ages between 60 and 75 years old (*p* < 0.001; OR = 6.3[2.1–18.9]) and abdominal origin bacteraemia (*p* = 0.02; OR = 5[1.2–20.5]) were risk factors for the severity of the infection (Table 2).

**Table 2:** Risk factor death from *E. coli* bacteremia identified by univariate analyses.

**Risk factor**	Patients died^*^(n = 8)	**Other patients *(n = 40)***	**OR (95% CI)**	***p-value***
**CLINICAL, *n* (%)**				
60–75 years old	5 (62.5)	9 (22.5)	6.25 (2.1–18.3)	<0.001
Abdominal origin	3 (37.5)	3 (7.5)	5 (1.2–20.5)	0.021
**VIRULENCE FACTORS, *n* (%)**				
*iro*N	–	18 (45.0)	0.50 (0.42–0.73)	0.018
*cvaC*	–	15 (37.5)	0.63 (0.49–0.80)	0.044
*afa*	3 (37.5)	2 (5.0)	11.4 (1.5–85.7)	0.027

*Chi square test. Data expressed as absolute number (%)*.

* *Patients died due to E. coli bacteraemia*.

### Antibiotic Susceptibility

**Figure 1:**
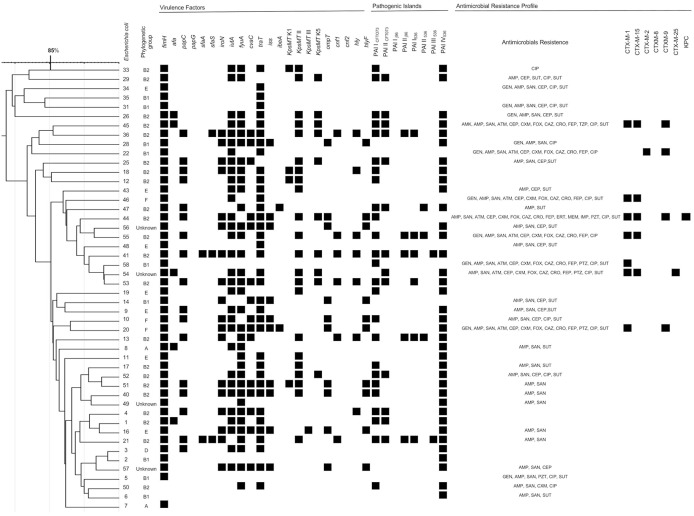
ERIC-PCR molecular fingerprint profiles of 48 *Escherichia coli* bloodstream infection isolates, virulence determinants and antimicrobial resistance profiles. Amikacin (AMK), gentamicin (GEN), ampicillin (AMP), ampicillin-sulbactam (SAN), aztreonam (ATM), cephalothin (CEP), cefuroxime (CXM), cefoxitin (FOX), ceftazidime (CAZ), ceftriaxone (CRO), cefepime (FEP), imipenem (IMP), meropem (MEM), ertapenem (ERT), piperacillin-tazobactan (PTZ), ciprofloxacin (CIP), and sulfamethoxazole.

The *E. coli* isolates exhibited a high rate of resistance to ampicillin (64.6%), ampicillin-sulbactam (56.3%), sulfamethoxazole-trimethoprim (45.8%), and ciprofloxacin (35.4%). Nine (18.8%) isolates were resistant to gentamicin, 1 (2.1%) was resistant to amikacin, 7 (14.6%) were resistant to piperacillin-tazobactam and only 1 (2.1%) isolate was resistant to carbapenems and was positive for *bla*_KPC_ (Figure 1).

The CTX-M enzymes are important β-lactamases described in worldwide. In this study, 48 isolates 8 (16.7%) were resistant to third and fourth generation cephalosporin and were producing ESBL type CTX-M. Seven (14.6%) isolates were positive for CTX-M-1, and five isolates were positive for CTX-M-15. Other variants were also found, including CTX-M-9, CTX-M-2, and CTX-M-25. All ExPEC-producing CTX-M showed a combination of more than one enzyme except for 1 isolate (Figure 1).

### Phylogenetic Analysis

The phylogenetic group B2 (45.8%) was predominant among the ExPEC strains. Groups B1 (18.8%), E (14.6%), F (6.3%), A (4.2%), and D (2.1%) exhibited the lowest frequencies, and 4 (8.2%) isolates were not grouped.

### Distribution of Virulence Factors and Pathogenicity Islands

In the present study, the most predominant virulence genes were *fim*H (95.8%), *tra*T (77.1%), *fyu*A (70.8%), *iut*A (64.3%), *kpsMT* II (45.8%), *iro*N (37.5%), *pap*C (35.4%), *cva*C (31.3%), *iss* (20.8%), *kpsMT* K5 (20.8%), *omp*T (20.8%), *hly*F (20.8%), *hly*A (14.6%), *cnf* 1 (12.5%), *afa* (10.4%), *kpsMT* K1 (8.3%), *sfa*S (6.3%), *sfa*A (4.2%), *ibe*A (4.2%), and *kpsMT* III (2.1%). No isolates were positive for the genes *pap*G and *cnf* 2 (Table 3).

**Table 3:** Prevalence of virulence factors, islands of pathogenicity of 48 blood *Escherichia coli* isolates, 2015–2018.

**Virulence factors**	**Gene**	***n* (%)**
Type 1 fimbrial adhesin	*fim*H	46 (95.8)
Outer membrane lipoprotein	*tra*T	37 (77.1)
Yersiniabactin siderophore receptor	*fyu*A	34 (70.8)
Aerobactin siderophore receptor	*iut*A	31 (64.3)
Group capsular II	*kps*MT II	22 (45.8)
Salmochelin siderophore receptor	*iro*N	18 (37.5)
P fimbriae C	*pap*C	17 (35.4)
Colicin C	*cva*C	15 (31.3)
Outer membrane protease	*omp*T	10 (20.8)
K5 capsule	*kps*MT K5	10 (20.8)
Haemolysin F	*hly*F	10 (20.8)
Increased serum survival	*iss*	10 (20.8)
Haemolysin A	*hly*A	7 (14.6)
Cytotoxic necrotizing factor 1	*cnf*1	6 (12.5)
Adhesin Afa	*afa*	5 (10.4)
K1 capsule	*kps*MT K1	4 (8.3)
S fimbriae (S)	*sfa*S	3 (6.3)
S fimbriae (A)	*sfa*A	2 (4.2)
Invasion of brain endothelium	*ibe*A	2 (4.2)
Group capsular III	*kps*MT III	1 (2.1)
Cytotoxic necrotizing factor 2	*cnf*2	-
P fimbriae (G)	*pap*G	-
**PATHOGENICITY ISLANDS**		
PAI IV_536_		37 (77.1)
PAI I_CFT073_		25 (52.1)
PAI II_CFT073_		13 (27.1)
PAI I_536_		6 (12.5)
PAI II_J96_		5 (10.4)
PAI II_536_		3 (6.3)
PAI III_536_		2 (4.2)
PAI I_J96_		-
PAI I_J96_		

The presence of the *afa* gene was associated with mortality due to bacteraemia caused by *E. coli* (*p* = 0.027; OR = 11.4[1.5–85.7]). This is unlike the *iro*N (*p* = 0.018; OR = 0.50[0.42–0.73]) and *cva*C genes (*p* = 0.044; OR = 0.63[0.49–0.80]), which were not present in the same isolates (Table 2).

Ten (20.8%) isolates were positive for the *cva*C, *hly*F, *iro*N, *iss*, and *omp*T genes. These genes have been described as the conserved virulence plasmidic (CVP) region (having 8 genes or operons: *iro, iuc, sit, omp*Tp, *cva, hly*F, *ets*, and *iss*). Six of these isolates were classified into 4 different phylogenetic groups (B2, B1, F, and E), and two isolates were not grouped.

The most prevalent PAI was PAI IV_536_ (77.1%); this was followed by PAI I_CFT073_ (52.1%), PAI II_CFT073_ (27.1%), PAI I_536_ (12.5%), PAI II_J96_ (10.4%), PAI II_536_ (6.3%), and PAI III_536_ (4.2%). PAI I_J96_ was not detected in this study (Table 2). The bloodstream isolates contained at least one of the investigated PAIs, and the most frequent combination pattern was related to those strains with PAI IV_536_ and PAI I_CFT073_ (47.9%). No strain presented all the PAIs.

### Comparison of Virulence Factors and Pathogenicity Islands With Phylogroups

The phylogenetic group B2 had the highest frequency regarding both the virulence genes and PAI markers compared those in the other phylogenetic groups. In this investigation, there was a significant association between the phylogenetic group B2 and the presence of virulence genes such as *pap*C, *fyu*A, *kpsMT* K1, *kpsMT* K5, *kpsMT II, hly*A, and *cnf* 1 (Table 4).

**Table 4:** Phylogenetic group relation with virulence factors and islands of pathogenicity of 48 blood *Escherichia coli* isolates, 2015–2018.

**Virulence factor, *n* (%)**	**Gene**	**Phylogenetic groups**		**Total (*n* = 48)**	***p*-value**
		**B2 (*n* = 22)**	**Non-B2 (*n* = 26)**		
Type 1 fimbrial adhesin	*fim*H	21 (45.7)	25 (54.3)	46 (95.8)	-
Outer membrane lipoprotein	*tra*T	19 (51.4)	18 (48.6)	37 (77.1)	-
Yersiniabactin siderophore receptor	*fyu*A	21 (61.8)	13 (38.2)	34 (70.8)	0.001
Aerobactin siderophore receptor	*iut*A	17 (54.8)	14 (45.2)	31 (64.3)	-
Group capsular II	*kps*MT II	18 (81.8)	4 (18.2)	22 (45.8)	0.001
Salmochelin siderophore receptor	*iro*N	11 (61.1)	7 (38.9)	18 (37.5)	-
P fimbriae C	*pap*C	14 (82.4)	3 (17.6)	17 (35.4)	<0.001
Colicin C	*cva*C	8 (53.3)	7 (46.7)	15 (31.3)	-
Outer membrane protease	*omp*T	3 (30.0)	7 (70.0)	10 (20.8)	-
K5 capsule	*kps*MT K5	9 (90.0)	1 (10.0)	10 (20.8)	0.002
Haemolysin F	*hly*F	3 (30.0)	7 (70.0)	10 (20.8)	-
Increased serum survival	*iss*	3 (30.0)	7 (70.0)	10 (20.8)	-
Haemolysin A	*hly*A	7 (100.0)	-	7 (14.6)	0.002
Cytotoxic necrotizing factor 1	*cnf*1	6 (100.0)	-	6 (12.5)	0.004
Adhesin Afa	*afa*	3 (60.0)	2 (40.0)	5 (10.4)	-
K1 capsule	*kps*MT K1	4 (100.0)	-	4 (8.3)	0.023
S fimbriae (S)	*sfa*S	3 (100.0)	-	3 (6.3)	-
S fimbriae (A)	*sfa*A	2 (100.0)	-	2 (4.2)	-
Invasion of brain endothelium	*ibe*A	1 (50.0)	1 (50.0)	2 (4.2)	-
Group capsular III	*kps*MT III	-	1 (100.0)	1 (2.1)	-
Cytotoxic necrotizing factor 2	*cnf*2	-	-	-	-
P fimbriae (G)	*pap*G	-	-	-	-
**PATHOGENICITY ISLANDS, *n* (%)**				
PAI IV_536_	21(56.8)	16 (43.2)	37 (77.1)	0.005
PAI I_CFT073_	22 (88.0)	3 (12.0)	25 (52.1)	<0.001
PAI II_CFT073_	12 (92.3)	1 (7.7)	13 (27.1)	<0.001
PAI I_536_	6 (100.0)	-	6 (12.5)	0.010
PAI II_J96_	5 (100.0)	-	5 (10.4)	0.010
PAI II_536_	3 (100.0)	-	3 (6.3)	-
PAI III_536_	2 (100.0)	-	2 (4.2)	-
PAI I_J96_	-	-	-	-

*Chi square test. Data expressed as absolute number (%)*.

An increased presence of PAI markers was observed in the phylogenetic group B2, while the lowest presence of PAI markers was related to the other phylogroups. The high frequency of the B2 group was statistically significant in PAI I_536_, PAI IV_536_, PAI I_CFT073_, PAI II_CFT073_, and PAI II_J96_ (Table 4).

### Genetic Correlation Between *E. coli* Blood Strains

Analysis of molecular genotyping using the ERIC-PCR technique demonstrated the presence of two major clonal lineages and several isolates that were not related to each other (Figure 1). In the data analysis, a cutoff of 85% was used due to the high diversity of the isolates. A more detailed analysis verified that the two lineages were significantly different in relation to the antimicrobial resistance profile (Table 5). The differences were significant for ESBL production and for the presence of the *bla*_CTX−M−1_, *bla*_CTX−M−15_, and *bla*_CTX−M−9_ genes. In addition, the two clonal groups showed significant differences in a single VF, the *kpsMT* K5 gene. The most prevalent phylogenetic group remained the B2 group in the two clonal groups, and presented a similar prevalence in the other phylogroups (Table 6).

**Table 5:** Comparison of virulence factors, pathogenicity islands and resistance profile between two clonal groups.

**Genetic determinants**		**Clonal group 1 (*n* = 15)**	**Clonal group 2 (*n* = 8)**	**Total**	***p*-value**
**VIRULENCE FACTOR, *n* (%)**					
Type 1 fimbrial adhesin	*fim*H	13 (61.9)	8 (38.1)	21 (91.3)	-
Outer membrane lipoprotein	*tra*T	11 (61.1)	7 (38.9)	18 (78.3)	-
Yersiniabactin siderophore receptor	*fyu*A	12 (70.6)	5 (29.4)	17 (73.9)	-
Aerobactin siderophore receptor	*iut*A	8 (57.1)	6 (42.9)	14 (60.9)	-
Group capsular II	*kps*MT II	5 (50.0)	5 (50.0)	10 (43.5)	-
Salmochelin siderophore receptor	*iro*N	6 (60.0)	4 (40.0)	10 (43.5)	-
P fimbriae C	*pap*C	5 (55.6)	4 (44.4)	9 (39.1)	-
S fimbriae (A)	*sfa*A	5 (55.6)	4 (44.4)	9 (39.1)	-
Colicin C	*cva*C	5 (62.5)	3 (37.5)	8 (34.8)	-
Outer membrane protease	*omp*T	4 (66.7)	2 (33.3)	6 (26.1)	-
Haemolysin F	*hly*F	4 (66.7)	2 (33.3)	6 (26.1)	-
Increased serum survival	*iss*	4 (66.7)	2 (33.3)	6 (26.1)	-
K5 capsule	*kps*MT K5	1 (20.0)	4 (80.0)	5 (21.7)	0.016
Haemolysin A	*hlyA*	1 (25.0)	3 (75.0)	4 (17.4)	-
Cytotoxic necrotizing factor 1	*cnf*1	1 (25.0)	3 (75.0)	4 (17.4)	-
Adhesin Afa	*afa*	1 (50.0)	1 (50.0)	2 (8.7)	-
S fimbriae (S)	*sfa*S	1 (50.0)	1 (50.0)	2 (8.7)	-
K1 capsule	*kps*MT K1	1 (100.0)	-	1 (4.3)	-
Group capsular III	*kps*MT III	1 (100.0)	-		-
Invasion of brain endothelium	*Ibe*10	-	-	-	-
Cytotoxic necrotizing factor 2	*cnf*2	-	-	-	-
P fimbriae (G)	*pap*G	-	-	-	-
**PATHOGENICTY ISLANDS, *n* (%)**					
PAI IV_536_		14 (73.7)	5 (26.3)	19 (82.6)	-
PAI I_CFT073_		8 (68.7)	5 (31.3)	13 (56.5)	-
PAI II_CFT073_		4 (57.1)	3 (42.9)	7 (30.4)	-
PAI I_536_		1 (25.0)	3 (75.0)	4 (17.4)	-
PAI II_J96_		1 (33.3)	2 (66.7)	3 (13.0)	-
PAI II_536_		1 (50.0)	1 (50.0)	2 (8.7)	-
PAI III_536_		1 (100.0)	-	1 (4.3)	-
PAI I_J96_		-	-	-	-
**RESISTANCE PROFILE, *n* (%)**					
ESBL		-	4 (100.0)	4 (17.4)	0.003
CTX-M-1		-	4 (100.0)	4 (17.4)	0.003
CTX-M-15		-	3 (100.0)	3 (13.0)	0.006
CTX-M-9		-	1 (100.0)	1 (4.3)	-
CTX-M-25		-	1 (100.0)	1 (4.3)	-
KPC		-	1 (100.0)	1 (4.3)	-

*Chi-square Tess. Data expressed as absolute number (%)*.

**Table 6:** Phylogenetic classification among isolates belonging to clonal groups.

	**Phylogenetic groups**					
	**B1**	**B2**	**D**	**E**	NG^*^	***p-value***
Clonal group 1	3 (20.0%)	8 (53.3%)	1 (6.7%)	1 (6.7%)	2 (13.3%)	>0.05
Clonal group 2	3 (37.5%)	4 (50.0%)	-	1 (12.5%)	2 (25.5%)	>0.05

*Chi-square Test. Data expressed as absolute number (%)*.

* *NG (Not grouped)*.

## Discussion

### Demographic and Clinical Characteristics

ExPEC is a very important bacterium that is involved in bloodstream infections, and in severe cases, it may lead to death (Owrangi et al., 2018). For the characterization of this infection, it is necessary to consider the bacterial determinants, such as virulence factors, as well as antimicrobial susceptibility and host determinants (Jauréguy et al., 2007; Lefort et al., 2011). The understanding and characterization of these factors may facilitate the development of new strategies for the treatment of *E. coli* bloodstream infections.

The presence of intestinal diseases was very prevalent among patients (25.0%). It is possible for ExPEC to initiate systemic infections by causing sepsis or bacteraemia through abdominal translocation in patients with some intestinal disease (Vaishnavi, 2013). Diabetes was also a common comorbidity and was observed with an 18.8% prevalence. Studies have reported an association between diabetes and pyelonephritis by *E. coli* and the increased risk of complications such as renal abscess and necrosis (Alves et al., 2012).

These infections usually occur as complications of infections of the urinary or gastrointestinal tract or as complications of other infections (Micenková et al., 2017). In this study, 24 (50.0%) of the bloodstream infections had a urinary origin and 6 (12.5%) had an abdominal origin. Five of 6 patients with bactaeremia of abdominal origin had some abdominal disease and one had hepatic cirrhosis. Patients with hepatic cirrhosis had a risk of bacteraemia due to an altered immune system and an increased bacterial translocation (Lefort et al., 2011), and 8 of the 9 patients with diabetes had bacteraemia of a urinary origin.

The mortality associated with bacteraemia due to *E. coli* was 33.3%. According to Russo and Johnson (2003), *E. coli* bacteraemia has a case-fatality rate of 5 to 30% and represents an increasingly important endemic problem, accounting for hundreds of thousands of lives lost and billions of health care dollars spent each year. In agreement with the results of Lefort et al. (2011), a patient age between 60 and 75 years old (*p* < 0.001) was considered a risk factor for mortality. Additionally, as in this study, Lefort et al. (2011) found that the rate of death was higher among those whose sepsis originated from the digestive tract than it was for those whose sepsis originated from the urinary tract.

### Phylogenetic Classification, Virulence Factors and Pathogenicity Islands Distribution

Bacterial determinants were also important to characterize bacteraemia. The phylogenetic classification of ExPEC isolates showed that B2 (45.8%) and B1 (18.8%) were the most prevalent groups. In the literature, B2 was reported to be the most common phylogenetic group in ExPEC (Koga et al., 2014; Micenková et al., 2017). The presence of B1 isolates in bloodstream infections shows that this group is also capable of causing systemic infections; however, literature reports have stated that isolates that belong to the groups A and B1 are more often strictly commensal strains from the intestinal microbiota (Skjøt-Rasmussen et al., 2012; Usein et al., 2016).

According to Fratamico et al. (2016), groups B2 and D have a higher virulence in humans that allows them to induce extraintestinal infections in both healthy and immunocompromised hosts. However, in this study, a lower prevalence of group D was found. The low prevalence of *E. coli* from group D can be explained because in the old classification of Clermont et al. (2000), there was no distinction between groups D, E and F, and all strains were classified as group D.

One of the mechanisms responsible for the survival of *E. coli* strains in the bloodstream is the escape of the complement system through serum resistance (Miajlovic and Smith, 2014; Micenková et al., 2017). Multiple virulence factors have been shown to be involved in serum survival. In this study, 41 (85.4%) isolates of *E. coli* had one or more genes related to serum resistance, and the *tra*T gene presented a high prevalence among isolates (77.1%). Similar results were found in studies on *E. coli* bloodstream infections (Koga et al., 2014; Miajlovic et al., 2016; Bozcal et al., 2018). Among the capsules researched, group 2 (*kpsMT* II) was the most prevalent (45.8%) and encodes the K1 and K5 capsules (Russo et al., 1998); in addition, 10 (20.8%) isolates presented the K5-specific capsule. In total, 23 (47.9%) of the *E. coli* isolates showed at least one capsule.

These polysaccharide antigens have been shown to be important for resistance to host immune responses in systemic infections (Miajlovic and Smith, 2014). K5 is involved in the pathogenesis of extraintestinal infections, such as bacteraemia, urinary tract infections, and neonatal meningitis (Blundell et al., 2009). In addition, the K5 capsule confers resistance to both the human innate and adaptive immune system. The chemical structure is similar to heparan sulfate and is probably what contributes to the ability to escape from the immune system, thus, conferring high virulence to the *E coli* isolates (Blundell et al., 2009).

Iron is a necessary element in virtually all microorganisms and is utilized to catalyze a wide variety of indispensable enzymatic reactions, but iron is also essential to host cells. However, the affinities of bacterial siderophores to iron are generally much higher than those of host proteins/molecules, allowing pathogens to outcompete the host in iron acquisition (Wilson et al., 2017). These siderophore systems are commonly associated with ExPEC strains and those isolated from bacteraemia (Koga et al., 2014; Bozcal et al., 2018). In the present study, yersiniabactin (*fyu*A) was the most prevalent, and was present in 70.8% of the isolates tested. The *fyu*A gene product is involved in the efficient uptake of iron from the bloodstream (Ananias and Yano, 2008) and in invasion of the bloodstream from the urinary tract (Johnson and Stell, 2000). Lefort et al. (2011) reported results regarding *fyu*A and mortality, and found that *fyu*A was present more frequently in survivors (78.09 vs. 66.18%, *p* = 0.0025).

In this study, genes related to adhesins were researched and included type 1 fimbria (*fim*H), P fimbria (*pap*C and *pap*G), Dr binding adhesin (*afa*), and S fimbria (*sfa*A and *sfa*S). The most prevalent was *fim*H (95.8%). FimH is a crucial factor for the virulence of uropathogenic *E. coli* strains by mediating adhesion to uroepithelial proteins (Dale and Woodford, 2015) and bacteraemia usually occurs as a complication of an infections in urinary tract (Laupland, 2013). The second most prevalent adhesin gene was *pap*C, which is also involved in urinary tract infections and subsequent bacteraemia (Lefort et al., 2011; Subashchandrabose and Mobley, 2015). Ananias and Yano (2008) reported that the association of *pap*C with *fyu*A could be the minimal prerequisite for bacterial passage from a renal focus of infection into the bloodstream of non-compromised patients, attached to *kpsMT* II or to another capsule or protectin.

The genes related to toxin and haemolysin production were researched and included *cnf* 2, *cnf* 1, *hly*A, *hly*F, *cva*C, and *ibe*A. The *cva*C and *hly*A genes are frequently detected in ExPEC strains (Koga et al., 2014). The prevalence of colicin V (*cva*C) was 31.3%. Its role in the pathogenesis of bloodstream infections is not well-understood, but *cva*C inhibits the cell growth of other bacteria by, reducing competition for nutrients in conditions of scarcity or stress (Gérard et al., 2005); in addition, *cvaC* is present on the ColV plasmid, which is known to encode the aerobactin iron uptake system as well as serum resistance factors (Mokady et al., 2005). According to Nedialkova et al. (2014), colicin production is a common trait in *E. coli* populations and on average, 30% of natural *E. coli* populations produce one or more colicins.

The toxin α-haemolysin (*hly*A) was present in 14.6% of isolates. This toxin incites a noxious inflammatory response, thereby causing clinical disease. The *E. coli* protein HlyA is a pore-forming bacterial exotoxin that may contribute to the virulence of bacteria during bloodstream infections and sepsis (Sonnen and Henneke, 2013). Bonacorsi et al. (2006) found that in infants younger than 90 days, the presence of *hly*A and/or *iro*N, similar to the presence of *hly*A and/or antigen K1, was associated with strains causing urinary tract infections that lead to bacteraemia.

Virulence genes are mainly organized in large clusters called PAIs or are plasmids that have integrated into the genome and, by horizontal gene transfer, explain the notable plasticity of the *E. coli* genome (Phillips-Houlbracq et al., 2018). Thirty-nine (81.3%) of the isolates were positive for one or more PAI, and the most prevalent was PAI IV_536_ (77.1%). This PAI is considered an island of high pathogenicity and is associated with the synthesis and transport of the yersiniabactin siderophore, one of the main bacterial iron uptake systems (Dobrindt et al., 2002). This information was confirmed by the presence of the *fyu*A gene in 70.8% of isolates, and all the isolates that were positive for *fyu*A were also positive for PAI IV_536_. PAIs were found primarily in the strains of uropathogenic *E. coli*, and the high prevalence among the *E. coli* strains isolated from bloodstream infections can be associated with the fact that urinary tract infections are very common type of infection, and bacteraemia is often a complication of this infection.

Ten (20.8%) isolates were positivite for the *cva*C, *hly*F, *iro*N, *iss*, and *omp*T genes. The presence of these genes and of the genes encoding salmochelin, aerobactin, and the iron–uptake system SitABC was a signature of conserved virulence plasmidic (CVP) (Lemaître et al., 2013). The phylogenetic classification of these isolates showed that commensal strains also have virulence determinates that cause extraintestinal infections. Only 3 out of the 10 isolates were classified in the B2 group.

Our results demonstrate that the group B2 had the highest frequency regarding both virulence genes and PAI markers compared to those in the non-B2 group. Three of the 4 polysaccharide capsules (*kpsMT* K1, *kpsMT* K5, and *kpsMT* II) studied were more prevalent in the isolates of group B2. The genes *pap*C, *fyu*A, *hly*A, and *cnf* 1 were also statistically more prevalent in group B2 isolates. Likewise, 5 out of the 8 PAIs studied were statistically more prevalent in the isolates of group B2. These results represent the ability of group B2 isolates to cause extraintestinal infections, such as infections of the bloodstream (Johnson and Russo, 2002; Köhler and Dobrindt, 2011).

### Molecular Genotyping and Resistance Profile

The analysis of molecular genotyping demonstrated the presence of two clonal lineages and several isolates not related to each other. A lineage composed of 8 isolates, of which, 7 were positive for the presence of the *bla*_CTX−M_ gene, and another lineage composed of 15 non-ESBL producing isolates. The results show no significant difference between the two strains in relation to the other antimicrobials tested or in relation to virulence determinates, except for *KpsMT* K5 which was more prevalent in the clonal lineage that presented a resistance profile to β-lactams. However, there was no relationship between clonality and clinical outcome (mortality due to bacteraemia caused by *E. coli*).

In this study, 16.7% (*n* = 8) of isolates were ESBL producers and were positive for the presence of the *bla*_CTXM_ gene. CTX-M enzymes have become the predominant ESBLs encountered in the clinic, and the rapid dissemination of ESBL is an alarming trend and is considered to be one of the world's main health threats (Yair and Gophna, 2018). It was previously reported that CTX-M-producing *E. coli* isolates often carry resistance to additional antibiotic classes, which can include co-trimoxazole, aminoglycosides, and fluoroquinolones (Pitout and Laupland, 2008). Six of the 8 isolates showed resistance to sulfamethoxazole-trimethoprim and aminoglycosides, and all isolates showed resistance to ciprofloxacin.

The prevalence of antimicrobial resistance is lower among strains belonging to the B2 phylogenetic group, suggesting a trade-off between resistance and virulence (Clermont et al., 2008). Our results show that only 2 of the 22 isolates classified into group B2 were ESBL producers and that 9 of 14 isolates, sensitive to all antibiotics, belonged to group B2. The rate of *E. coli* isolation from the bloodstream and the production of ESBL is quite variable. Miajlovic et al. (2016) found a prevalence of 40% of ESBL producers in Ireland; however, Burdet et al. (2014) and Namikawa et al. (2017) both found a lower prevalence: at 3.6% and at <10.0%, respectively. In Brazil, Seki et al. (2013) found a prevalence of 15% of ESBL producers, and 55% were producers of CTX-M-15. We found a similar prevalence: 16.6% were producers of ESBLs and 62.5% were producer of CTX-M-15.

In summary, these results indicate that the bloodstream infection process can be mediated by several alternative VFs, and each strain may have a unique combination of these factors. This variety of virulence genes can be explained by several genetic factors that contribute to genomic plasticity, such as plasmids, phages, and transposable elements. Multiple VFs were found however most of them in relatively small number of isolates, thus, the study did not have enough power to determine their effect on mortality. Although the phylogenetic group B2 corresponds with the virulence potential, our results show that isolates of the B1 (commensal) and E groups are also capable of causing extraintestinal infections, and the VFs carried by these strains appear to be more important in determining the pathogenic potential than is the organism's phylogenetic background. Other important factors to be considered in the pathogenesis of *E. coli* bloodstream infections are host characteristics, such as the portal of entry and comorbidities. Likewise, the resistance profile is important information for the management and treatment of patients with *E. coli* bloodstream infections. Among ESBL-producing *E. coli* isolates, the CTX-M-1 group (CTX-M-15) had a high prevalence, and some of the more recent resistance mechanisms, such as CTM-M-15, arose from high-risk clones that facilitate the persistence and spread of resistance around the world. This study is important because it shows the clinical and molecular characteristics of *E. coli* bloodstream infections from Brazilian isolates.

## Limitation

The work was performed using the PCR and ERIC-PCR techniques. Further sequencing of *E. coli* strains causing bacteraemia could aid in the finding and description of genes or gene clusters that are peculiar to the *E. coli* strains that cause bacteremia.

## Data Availability

The datasets generated for this study are available on request to the corresponding author.

## Author Contributions

AD, EV, and RK: conceived and designed the experiments. AD and JS: performed the experiments. AD, EV, and RK: analyzed the data. RK and VK: contributed reagents and materials. MP and MREP: reviewed the study. CdM: performed confirmation experiments.

### Conflict of Interest Statement

The authors declare that the research was conducted in the absence of any commercial or financial relationships that could be construed as a potential conflict of interest.
