# Supplementary material for: Escherichia coli Bloodstream Infections in Patients at a University Hospital: Virulence Factors and Clinical Characteristics
Source: Front Cell Infect Microbiol. 2019 Jun 6;9:191. doi: 10.3389/fcimb.2019.00191 (PMC6563721; doi:10.3389/fcimb.2019.00191)
Supplement: Supplementary file 1 [file Table_1.docx]

Supplementary Material

| **Table S1.** Genes encoding the ESBL-CTXM and KPC enzymes, oligonucleotide sequence and amplified fragment size | | | | |
| --- | --- | --- | --- | --- |
| **Enzymes** | **Gene** | **Prime sequence (5–3)** | **Amplic size (bp)** | **References** |
| CTX-M-1 | *bla*_CTX-M1_ | AAA AAT CAC TGC GCC AGT TC | 415 | Woodford et al., 2006 |
|  |  | AGC TTA TTC ATC GCC ACG TT |  |  |
| CTX-M-2 | *bla*_CTX-M2_ | CGA CGC TAC CCC TGC TAT T | 552 | Woodford et al., 2006 |
|  |  | CCAGCGTCAGAT TTT TCA GG |  |  |
| CTX-M-8 | *bla*_CTX-M8_ | TCG CGT TAA GCG GAT GAT GC | 666 | Woodford et al., 2006 |
|  |  | AAC CCA CGA TGT GGG TAG C |  |  |
| CTX-M-9 | *bla*_CTX-M9_ | CAA AGA GAG TGC AAC GGA | 205 | Woodford et al., 2006 |
|  |  | ATT GGA AAG CGT TCA TCA CC |  |  |
| CTX-M-25 | *bla*_CTX-M25_ | GCA CGA TGA CAT TCG GG | 327 | Woodford et al., 2006 |
|  |  | AAC CCA CGA TGT GGG TAG C |  |  |
| CTX-M-15 | *bla*_CTX-M15_ | ATA AAA CCG GCA GCG GTG | 483 | Leflon-Guibout et al., 2004 |
|  |  | GAA TTT TGA CGA TCG GGG |  |  |
| KPC | *bla*_KPC_ | ATG TCA CTG TAT CGC CGT CT | 850 | Bradford et al., 2004 |
|  |  | TTT TCA GAG CCT TAC TGC CC |  |  |

| **Table S2.** Genes researched for phylogenetic classification, with their respective primers and nucleotide sequence. | | | | |
| --- | --- | --- | --- | --- |
| **PCR reaction** | **Genes** | **Prime sequence (5–3)** | **Amplic size (bp)** | **Reference** |
| **Multiplex** | *chu*A | GAC GAA CCA ACG GTC AGG AT | 279 | Clermont et al., 2013 |
|  |  | TGC CGC CAG TAC CAA AGA CA |  |  |
|  | *yj*aA | TGA AGT GTC AGG AGA CGC TG | 211 |  |
|  |  | ATG GAG AAT GCG TTC CTC AAC |  |  |
|  | TSPE4.C2 | GAG TAA TGT CGG GGC ATT CA | 152 |  |
|  |  | CGC GCC AAC AAA GTA TTA CG |  |  |
| **Grupo E** | *arp*A | GAT TCC ATC TTG TCA AAA TAT GCC | 219 | Clermont et al., 2013 |
|  |  | GAA AAG AAA AAG AAT TCC CAA GAG |  |  |
| **Grupo C** | *trp*A | AGT TTT ATG CCC AGT GCG AG | 489 | Clermont et al., 2013 |
|  |  | TCT GCG CCG GTC ACG CCC |  |  |

| **Table S3.** Genes encoding virulence factors, oligonucleotide sequence and amplified fragment size. | | | | |
| --- | --- | --- | --- | --- |
| **Genes** | **Prime sequence (5–3)** | **Coded virulence factors** | **Amplic size (bp)** | **References** |
| *kpsMT* II | GCG CAT TTG CTG ATA CTG TTG | Group 2 of capsular antigens | 272 | Johnson and Stell, 2000 |
|  | CAT CCA GAC GAT AAG CAT GAC CA |  |  |  |
| *KpsMT* III | TCC TCTT GCT ACT ATT CCC CCT | Group 3 of capsular antigens | 392 | Johnson and Stell, 2000 |
|  | AGG CGT ATC CAT CCC TCC TAA C |  |  |  |
| *KpsMT* k1 | TAG CAA ACG TTC TAT TGG TGC | K1 capsule | 153 | Johnson and Stell, 2000 |
|  | CAT CCA GAC GAT AAG CAT GAC CA |  |  |  |
| *kpsMT* k5 | CAG TAT CAG CAA TCG TTC TGT A | K5 capsule | 159 | Johnson and Stell, 2000 |
|  | CAT CCA GAC GAT AAG CAT GAC CA |  |  |  |
| *cva*C | CAC ACA CAA ACG GGA GCT GTT | Colicin V | 680 | Johnson and Stell, 2000 |
|  | CTT CCC GCA GCA TAG TTC CAT |  |  |  |
| *iut*A | GGC TGG ACA TCA TGG GAA CTG G | Aerobactin  siderophore receptor | 300 | Johnson and Stell, 2000 |
|  | CGT CGG GAA CGG GTA GAA TCG |  |  |  |
| *fim*H | TGC AGA ACG GAT AAG CCG TGG | Fimbriae type 1 | 508 | Johnson and Stell, 2000 |
|  | GCA GTC ACC TGC CC TCC GGT A |  |  |  |
| *fyu*A | TGA TTA ACC CCG CGA CGG AA | Yersiniobactin siderophore receptor | 880 | Johnson and Stell, 2000 |
|  | CGC AGT AGG CAC GAT CTT GTA |  |  |  |
| *pap*C | GAC GGC TGT ACT GCA GGG TGT GGC G | P Fimbriae | 328 | Johnson and Stell, 2000 |
|  | ATA TCC TTT CTG CAG GCA GGG TGT GGC |  |  |  |
| *pap*G | CTG TAA TTA CGG AAG TGA TTT CTG | P Fimbriae | 1.070 | Johnson and Stell, 2000 |
|  | CTG TAA TTA CGG AAG TGA TTT CTG |  |  |  |
| *sfa*A | CTC CGG AGA ACT GGG TGC ATC TTA C | Sfa fimbriae | 410 | Johnson and Stell, 2000 |
|  | CGG AGG AGT AAT TAC AAA CCT GGC A |  |  |  |
| *sfa*S | GTG GAT ACG ACG ATT ACT GTG | Sfa fimbriae | 240 | Johnson and Stell, 2000 |
|  | CCG CCA GCA TTC CCT GTA TTC |  |  |  |
| *afa* | GGC AGA GGG CCG GCA ACA GGC | Afa fimbriae | 750 | Johnson and Stell, 2000 |
|  | CCC GTA ACG CGA CAG CAT CTC |  |  |  |
| *ibe*A | AGG CAG GTG TGC GCC GCG TAC | Invasion of brain endothelium | 170 | Johnson and Stell, 2000 |
|  | TGG TGC TCC GGC AAA CCA TGC |  |  |  |
| *Hly* | AAC AAG GAT AAG CAC TGT TCT GGC | Hemolysin | 1.177 | Johnson and Stell, 2000 |
|  | ACC ATA TAA GCG GTC ATT CCC GTC |  |  |  |
| *cnf*1 | AGG AAG TTA TAT TTC CGT AGG | Cytotoxic necrotizing factor 1 | 498 | Johnson and Stell, 2000 |
|  | GTA TTT GCC TGA ACC GTA A |  |  |  |
| *cnf*2 | AAT CTA ATT AAA GAG AAC | Cytotoxic necrotizing factor 2 | 543 | Johnson and Stell, 2000 |
|  | CAT GCT TTG TAT ATC TA |  |  |  |
| *tra*T | GGT GTG GTG CGA TGA GCA CAG | Serum resistance | 290 | Johnson and Stell, 2000 |
|  | GGT GTG GTG CGA TGA GAC CAG |  |  |  |
| *iro*N | AAT CCG GCA AAG AGA CGA ACC GCC T | Salmochelin siderophore receptor | 553 |  |
|  | GTT CGG GCA ACC CCT GCT TTG ACT TT |  |  |  |
| *omp*T | TCA TCC CGG AAG CCT CCC TCA CTA CTA T | Episomal outer membrane protease | 496 | Johnson and Stell, 2000 |
|  | TAG CGT TTG CTG CAC TGG CTT CTG ATA C |  |  |  |
| *hly*F | GGC CAC AGT CGT TTA GGG TGC TTA CC | Putative avian hemolysin | 450 | Johnson and Stell, 2000 |
|  | GGC GGT TTA GGC ATT CCG ATA CTC AG |  |  |  |
| *Iss* | CAG CAA CCC GAA CCA CTT GAT G | Episomal increased serum survival | 323 | Johnson and Stell, 2000 |
|  | AGC ATT GCC AGA GCG GCA GAA |  |  |  |

| **Table S4**. Genes encoding islands of pathogenicity, oligonucleotide sequence and size of the amplified fragment | | | | |
| --- | --- | --- | --- | --- |
| **Pathogenicity Islands** | **Primer name** | **Prime sequence (5–3)** | **Amplic size (bp)** | **Reference** |
| **PAI I_536_** | I.9 | TAA TGC CGG AGA TTC ATT GTC | 1.800 | Koga et al., 2014 |
|  | I.10 | AGG ATT TGT CTC AGG GCT TT |  |  |
| **PAI II_536_** | orf1up | CAT GTC CAA AGC TCG AGC C | 1.000 | Sabaté et al., 2006 |
|  | orf1down | CTA CGT CAG GCT GGC TTT G |  |  |
| **PAI III_536_** | sfaAI.1 | CGG GCA TGC ATC AAT TAT CTT TG | 161 | Sabaté et al., 2006 |
|  | sfaAI.2 | TGT GTA GAT GCA GTC ACT CCG |  |  |
| **PAI IV_536_** | IRP2 FP | AAG GAT TCG CTG TTA CCG GAC | 300 | Sabaté et al., 2006 |
|  | IRP2 RP | TCG TCG GGC AGC GTT TCT TCT |  |  |
| **PAI I_CFT073_** | RPAi | GGA CAT CCT GTT ACA GCG CGC A | 930 | Sabaté et al., 2006 |
|  | RPAf | TCG CCA CCA ATC ACA GC GAA C |  |  |
| **PAI II_CFT073_** | cft073.2Ent1 | ATG GAT GTT GTA TCG CGC | 400 | Sabaté et al., 2006 |
|  | cft073.2Ent2 | ACG AGC ATG TGG ATC TGC |  |  |
| **PAI I_J96_** | papGIf | TCG TGC TCA GGT CCG GAA TTT | 400 | Sabaté et al., 2006 |
|  | papGIr | TGG CAT CCC ACA TTA TCG |  |  |
| **PAI II_J96_** | hlyd | GGA TCC ATG AAA ACA TGG TTA ATG GG | 2.300 | Sabaté et al., 2006 |
|  | cnf | GAT ATT TTT GTT GCC ATT GGT TAC C |  |  |
